# King Edward's Hospital Fund for London

**Published:** 1906-12-22

**Authors:** 


### HOSPITAL ADMINISTRATION.

### CONSTRUCTION AND ECONOMICS.

### KING EDWARD'S HOSPITAL FUND FOR LONDON.

TO BE INCORPORATED BY SPECIAL ACT.

YEAR'S AWARDS TO HOSPITALS AND CONVALESCENT INSTITUTIONS.

A meeting of the General Council of King Edward's Hospital Fund for London, for the purpose of awarding grants to the hospitals and convalescent homes for the present year, was held on the 17th inst., at Marlborough House, his Royal Highness the Prince of Wales being in the chair. Those present were: The Lord Lieutenant of London (the Duke of Fife), the Bishop of London, the Chief Rabbi (Dr. Adler), Viscount Iveagh, Lord Rothschild, Lord Farquhar, the President of the Hospital Sunday and Hospital Saturday Funds (the Lord Mayor), the Chairman of the County Council (Mr. Evans Spicer), the President of the Royal College of Surgeons (Mr. Henry Morris), Sir Savile Crossley, Sir Joseph Dimsdale, Mr. Stuart-Wortley, K.C., M.P., Sir Trevor Lawrence, Sir W. S. Church, Sir Julius Wernher, Sir Edgar Speyer, Sir Henry Burdett, Sir W. J. Collins, M.P., Sir John Craggs, Mr. Frederick M. Fry, Mr. William Latham, K.C., Mr. J. Danvers Power, Mr. Albert G. Sandeman, and Mr. Hugh C. Smith. Letters of regret were reported from the following: The Lord Lieutenant of Middlesex (the Duke of Bedford), the Duke of Norfolk, the Bishop of Southwark, Archbishop Bourne, the Rev. Dr. Guinness Rogers, the Rev. Dr. Stevenson, the Earl of Bessborough, Lord Strathcona and Mount Royal, the President of the Royal College of Physicians (Sir R. Douglas Powell), Sir John Aird, Bart., and Mr. J. Pierpont Morgan, jun.

Lord Rothschild reported that the amount received by the Fund to December 12, after payment of expenses, was £89,915, and in addition legacies to the amount of £35,000 had been declared, but not paid.

Sir Henry Burdett reported that the contribution from the League of Mercy would be £18,000, and said that the Home Counties had shown a great advance, and that the four counties, under the Presidency of Princess Victoria of Schleswig-Holstein, had sent the largest contribution from any President of the League.

The Chairman of the Executive Committee (Mr. Hugh Colin Smith) read the report of the Executive Committee as follows:—

"The Executive Committee recommend this year the distribution of £110,000 to the London hospitals, making, with the £1,000 entrusted to the Fund by the London Parochial Charities, for the Convalescent Homes, a total distribution of £111,000. At the time of determining the amount for distribution legacies of £35,000 had been announced but not paid, and it was impossible to say whether they would be received before December 31 or not. The Committee, however, have no doubt that, having regard to the average receipts from legacies and to the amount carried forward last year, the sum of £111,000 can safely be distributed. The Executive Committee have adopted the reports of the Hospitals Distribution Committee and the Convalescent Homes Distribution Committee."

#### Distribution of the Fund.

The Chairman of the Hospitals Distribution Committee (Sir William Church) read the report of that committee as follows:—

"1. The committee have the pleasure to report that, with the approval of his Royal Highness the President, the sum of £110,000 was placed in their hands for distribution this year amongst the London hospitals, as against £100,000 in 1905.

"2. The number of hospitals applying for grants is 105, as against 106 last year, five having fallen out and four new applications having been received.

"3. The committee are pleased to learn that the City Orthopædic Hospital has now agreed to amalgamate with the Royal National Orthopædic Hospital, and that the arrangements are in course of being carried out. The sum of £250, voted conditionally last year to the City Orthopædic Hospital, remains in hand, and to this the committee have added £250, so that the assets of the hospital to be pooled on amalgamation will be increased by £500.

"4. In regard to the Throat, Nose, and Ear Hospitals, the committee are informed that the negotiations for amalgamation are proceeding. Towards this object the Distribution Committee, on the advice of the Committee appointed to deal with the matter, have set on one side £1,000 instead of making separate grants to each hospital. This grant is in addition to the considerable sums already promised by the Fund.

"5. The sum of £4,500 voted towards the removal of King's College Hospital to South London increases the total amount contributed by the Fund to this object to £16,000.

"6. The committee have this year continued the policy begun in 1905 of making exceptional grants in a few special instances. These amounts will be regarded in the nature of advances, and will, of course, be taken into consideration in the future. The exceptional grants are £5,000 to the Hampstead General Hospital, to enable it to take advantage of a conditional offer of £20,000; and £4,500 to the Mount Vernon Hospital for Consumption, to extinguish their debt on the buildings which are now completed.

"7. The committee recommend that grants made towards specified objects, such as building or other improvements, should in future be retained by the Fund until required. Several cases have been brought to the notice of the committee where large sums which have been granted in this way still remain unexpended. It seems desirable that some limit should be placed on the length of time during which such grants should be allowed to remain unexpended, and that they should be reconsidered every two years while they remain unapplied.

"8. It will be seen that in accordance with the committee's recommendation last year the distinction between annual grants and donations has been abolished.

"9. The attention of the committee has been called to the extensive arrangements for teaching nursing at hospitals; and while not wishing in any way to disparage work so obviously advantageous to the public, it may become necessary to consider to what extent expenditure is incurred for nursing beyond the requirements of the nursing service of each hospital.

"10. The subject of overbuilding, especially by institutions which are already unable to maintain their existing beds, has been considered by the Committee. They observe that the Council in 1903 sanctioned the following resolution of the Executive Committee: 'That in future any new hospital or those reconstructing or extending to a considerable extent within the area dealt with by the Fund be requested before taking definite action to submit their proposals to the Fund.' The committee recommend that the full purport and intention of this resolution should be brought each year to the notice of the hospitals.

"11. The reports of the visitors have as usual received the careful consideration of the committee and have been of the utmost value.

"12. The committee are pleased to observe considerable reduction in the cost per bed in a number of hospitals where the expenditure was formerly considered too high. Though much still remains to be done, the committee commend to public attention the general efforts being now made by the hospitals in the direction of economy.

"13. In addition to their published remarks the committee have followed and extended their usual practice in recommending that various suggestions, including many from the visitors' reports, should be made privately to some of the hospitals.

"The details of the awards to the various hospitals are appended."

### Awards to Hospitals.

Sir Savile Crossley presented the list of awards, as follows: The Distribution Committee draws attention to the fact that the absence of a grant does not necessarily imply dissatisfaction:—

Alexandra Hospital for Children.—£500, of which £100 for structural improvements.

Belgrave Hospital.—£1,250, of which £750 to reduce debt.

Billingsgate Mission Hospital.—The committee cannot view with approval the conversion of a dispensary into a hospital in a neighbourhood already well served with other institutions.

Blackheath and Charlton Cottage Hospital.—£75.

Bolingbroke Hospital.—£2,500, of which £2,250 to building fund.

British Lying-in Hospital.—£300, of which £100 to reduce debt.

Cancer Hospital.

Central London Ophthalmic Hospital.

Central London Throat and Ear Hospital.—See paragraph 4 of report.

Charing Cross Hospital.—£2,750. The committee make this grant in view of the new management. They, however, call attention to the fact that in adding forty beds to those already in use the former management seemed to ignore the fact that when there were in the hospital forty fewer beds than there now are its income failed to meet its expenditure.

Chelsea Hospital for Women.—£400.

Cheyne Hospital for Sick and Incurable Children.—£75, in consideration of the fact that some curable cases are admitted.

City of London Hospital for Diseases of the Chest, Victoria Park.—£2,250.

City of London Lying-in Hospital.—£1,000 for building.

City Orthopædic Hospital.—£250. The committee are glad to know that the hospital has now agreed to amalgamation, and £250 has been voted this year and added to the similar sum voted last year and retained in hand. This is in addition to the amount previously voted as the basis of the amalgamation scheme.

Clapham Maternity Hospital.—£100.

Dreadnought Hospital.—£3,000, of which £2,000 to improvements. The committee view with satisfaction the economical management of this hospital.

East End Mothers' Home.—£300.

East London Hospital for Children.—£2,000, of which £1,000 to improvements.

Eltham and Mottingham Cottage Hospital.—£50.

Evelina Hospital.—£1,100, of which £1,000 to out-patient department.

French Hospital.—£200.

Friedenheim Hospital.—£50.

General Lying-in Hospital.—£150.

German Hospital.—£200.

Gordon Fistula Hospital.—£25.

Great Northern Central Hospital.—£1,500.

Grosvenor Hospital for Women and Children.—£200.

Guy's Hospital.—£8,000. The committee view with satisfaction the continued economical management.

Hampstead General Hospital.—£5,000 to enable the hospital to take advantage of a conditional offer of £20,000 to complete the new building, for the maintenance of which continuous efforts to increase the annual revenue will be necessary.

Home and Infirmary for Sick Children.—£200, of which £50 towards building.

Home for Consumptive Females.—The committee consider that this home does not come within the description of a hospital as understood by the Fund.

Hospital for Consumption.—£2,500. The committee view with satisfaction the large reduction in the expenditure per bed.

Hospital for Diseases of the Throat.—See paragraph 4 of report.

Hospital for Epilepsy and Paralysis.—£450.

Hospital for Invalid Gentlewomen.—£200.

Hospital for Sick Children.—£1,500.

Hospital for Women.—£3,000, of which £2,000 to building fund.

Hospital of St. John and St. Elizabeth.—£500.

Infants' Hospital.—The committee do not regard this institution as a hospital in the sense accepted by the Fund.

Italian Hospital.—£500.

Kensington Dispensary and Children's Hospital.—£25.

Kensington General Hospital (late Queen's Jubilee Hospital).

King's College Hospital.—£6,000, of which £4,500 to Removal Fund.

London Fever Hospital.—£150.

London Homœopathic Hospital.—£500.

London Hospital.—£8,500. The committee view with satisfaction the considerable reduction in the cost per bed.

London Lock Hospital.—£1,500, of which £1,000 to nursing accommodation. The committee will be prepared to recommend a substantial grant towards the expense of rebuilding the Dean Street Hospital when a definite scheme has been prepared.

London Temperance Hospital.—£1,500, of which £1,000 to out-patient department.

London Throat Hospital.—See paragraph 4 of report.

Maternity Charity and District Nurses' Home, Plaistow.—£50.

Medical Mission Hospital (formerly entered as Cottage Hospital, Canning Town), Balaam Street, Plaistow.—£250 towards debt.

Memorial Cottage, Mildmay Park.—£50.

Metropolitan Hospital.—£2,000. The committee are pleased to see the successful efforts being made to reduce expenditure.

Middlesex Hospital.—£3,000. The committee are pleased to see the substantial economies effected during the period the wards were not closed for repairs.

Mildmay Mission Hospital.—£300.

Miller Hospital, Greenwich.—£1,250, of which £500 to extension.

Mount Vernon Hospital for Consumption (formerly entered as North London Hospital for Consumption).—£4,500, as a special donation this year in order to close the building debt.

National Anti-Vivisection Hospital.

National Dental Hospital.—£50, to reduce debt.

National Hospital for the Paralysed and Epileptic.—£1,750, of which £1,000 to reduce debt.

New Hospital for Women.—£500.

North-Eastern Hospital for Children.—£1,500, of which £500 to reduce debt. The committee are pleased to see that economies have been effected.

North-West London Hospital.—£500.

Norwood Cottage Hospital.—£50, towards improvements.

Oxygen Hospital.

Paddington Green Children's Hospital.—£400.

Passmore Edwards Acton Cottage Hospital.—£50.

Passmore Edwards East Ham Hospital.—£50.

Passmore Edwards Hospital for Willesden.—£25.

Passmore Edwards Cottage Hospital, Wood Green.

Poplar Hospital.—£500. The committee are glad to see the reduction in the total expenditure.

Queen Charlotte's Lying-in Hospital.—£500.

Royal Dental Hospital of London (formerly entered as Dental Hospital).—£500.

Royal Ear Hospital.—See paragraph 4 of report.

Royal Eye Hospital.—£400.

Royal Free Hospital.—£2,750.

Royal Hospital for Diseases of the Chest, City Road.—£400.

Royal London Ophthalmic Hospital.—£3,000. The committee consider that this hospital should receive more support from the public, and they trust that increased efforts will be made to secure such support.

Royal National Orthopædic Hospital.—£1,500. As agreed, upon satisfactory completion of amalgamation of the two hospitals. The committee are glad to learn that amalgamation with the City Orthopædic Hospital may now be considered as assured.

Royal Waterloo Hospital for Children and Women.—£1,500, of which £1,000 to building debt.

Royal Westminster Ophthalmic Hospital.—£100.

St. George's Hospital.—£2,000.

St. John's Hospital for Diseases of the Skin.—£100.

St. John's Hospital, Morden Hill, Lewisham.—£100.

St. Luke's House.—£25.

St. Mark's Hospital.—£100.

St. Mary's Hospital, Paddington.—£3,500. The Committee are pleased to see the considerable reduction in expenditure per bed.

St. Mary's Hospital for Women and Children, Plaistow.—£1,500, of which £1,000 for building.

St. Monica's Hospital.—£100.

St. Peter's Hospital for Stone.—£50.

St. Saviour's Hospital, Osnaburgh Street.—£200.

Samaritan Free Hospital.—£500.

Santa Claus Home.—£25, to improvements. In consideration of the fact that some curable cases are admitted.

South Wimbledon, Merton, and District Cottage Hospital.

Tottenham Hospital.—£2,000. The Committee view with satisfaction the economical management of this hospital.

University College Hospital.—£2,250.

Victoria Hospital for Children.—£1,100.

West End Hospital for Diseases of the Nervous System.—£100.

Western Ophthalmic Hospital.—£150, to reduce debt.

West Ham and East London Hospital.—£1,500, of which £1,000 to building. The Committee are pleased to see the reduction in expenditure.

West London Hospital.—£3,000, of which £1,500 for improvements.

Westminster Hospital.—£3,000, of which £2,000 towards debt.

Winifred House Invalid Children's Convalescent Nursing Home.

Woolwich and Plumstead Cottage Hospital.—£25.

Special Grant.—Set aside for amalgamation of Throat and Ear Hospitals, £1,000.

Summary.

| | |
|---|---|
| Grants ... ... ... ... ... | £109,000 |
| Special Grant set aside for amalgamation of Throat and Ear Hospitals | 1,000 |
| Total Grants to Hospitals for the year 1906 ... ... ... | £110,000 |

Convalescent Homes.

Awards.—The Chairman of the Convalescent Homes Committee (Mr. William Latham, K.C.) read the report of that Committee as follows :—

"Owing to the continued liberality of the trustees of the London Parochial Charities the Committee have again £1,000 to distribute.

"The Committee have continued their practice of giving the preference to institutions directly connected with London hospitals.

"The Committee have called the attention of the Executive Committee to the fact that the country branches of London consumption hospitals are not in strictness convalescent homes. At the same time, considering the nature of the malady, it is desirable that the treatment should be conducted outside the area hitherto visited by the visitors to hospitals."

The following awards are recommended :—

Alexandra Hospital, Convalescent Home at Painswick.—£25.

Charing Cross Hospital, Convalescent Home at Limpsfield, Surrey.—£50.

Chelsea Hospital for Women, Convalescent Home at St. Leonards.—£50.

East London Hospital for Children, Convalescent Home at Bognor.—£100.

Hospital for Sick Children, Convalescent Branch, Cromwell House, Highgate.—£25.

London Hospital, Convalescent Home at Tankerton, near Whitstable.—£50.

Mary Wardell Convalescent Home, Stanmore, Middlesex. £25.

Metropolitan Convalescent Institution, Convalescent Home at Walton.—£100.

Metropolitan Convalescent Institution, Convalescent Home at Broadstairs.—£100.

Middlesex Hospital, Convalescent Home at Clacton-on-Sea.—£200.

Mildmay Mission Hospital, Convalescent Home at Hendon. £50.

Mrs. Gladstone's Free Convalescent Home for the Poor, Ravensbury House, Mitcham, Surrey.—£25.

National Hospital for the Paralysed and Epileptic, Convalescent Home at East Finchley.—£25.

Paddington Green Children's Hospital, Convalescent Home at Slough.—£50.

Queen Charlotte's Lying-in Hospital, Convalescent Home at 20, Victoria Road, Kilburn.—£25.

Victoria Hospital for Children, Convalescent Home at Broadstairs.—£100.

Total Grants to Convalescent Homes, £1,000.

